# Supplementary material for: Decay of driver mutations shapes the landscape of intestinal transformation
Source: Nature. 2025 Dec 3;649(8097):729–38. doi: 10.1038/s41586-025-09762-w (PMC12804087; doi:10.1038/s41586-025-09762-w)
Supplement: Supplementary file 1 — Supplementary Tables 1–6 and Supplementary Figs. 1–6. [file 41586_2025_9762_MOESM1_ESM.pdf]

nature portfolio

https://doi.org/10.1038/s41586-025-09762-w

Supplementary information

Decay of driver mutations shapes the landscape of intestinal transformation

In the format provided by the authors and unedited

Supplementary Information

Decay of driver mutations shape the landscape of intestinal transformation

(Lourenço *et al.*)

Table of contents

Tables

Supplementary Table 1. Descriptive statistics of the Tam→ENU cohort.

Supplementary Table 2. Tamoxifen-only cohort descriptive statistics.

Supplementary Table 3. Sequencing gene panel.

Supplementary Table 4. dNdScv output table.

Supplementary Table 5. Global dN/dS estimates**.**

Supplementary Table 6. ENU→Tam cohort descriptive statistics.

Figures

Supplementary Fig. 1. Conditional Knock-out efficiency.

Supplementary Fig. 2. Mutation burden analysis.

Supplementary Fig. **3**. Copy number analysis in all cohorts.

Supplementary Fig. 4. Long term effects of ENU injection.

Supplementary Fig. **5**. Amplicon-seq coverage and allele frequencies.

Supplementary Fig. **6**. FACS gating strategy.

Cohort	n(Surv)	Surv	n(count)	Intestine	sd Intestine	SI	sd SI	proxSI	sd proxSI	distSI	sd distSI	Colon	sd Colon
Apc^{het}	5	78	3	1975.7	577.0	966.7	267.5	772.7	418.1	194.0	188.9	1009.0	484.9
$Arid1a^{null}$	9	312	6	1.8	1.2	0.3	0.5	0.3	0.5	0.0	0.0	1.5	0.8
Control	74	251	65	3.1	2.7	2.9	2.6	2.1	2.0	0.8	1.2	0.2	0.5
$Fbxw7^{null}$	14	64	11	157.3	41.1	156.9	40.9	103.9	17.2	53.0	26.1	0.4	0.5
$Kras^{G12D}$	10	37	3	920.7	184.5	388.0	126.8	352.3	105.4	35.7	22.7	532.7	77.5
$N1\text{-}ICD^{het}$	4	184	4	15.8	6.6	10.3	3.8	5.8	1.3	4.5	4.4	5.5	3.1
$Pik3ca^{H1047R}$	11	161	10	4.5	3.3	4.3	3.0	3.4	1.7	0.9	1.6	0.2	0.4
$Pten^{null}$	15	72	14	16.9	11.9	16.8	11.8	13.6	9.6	3.2	3.8	0.1	0.3
$Trp53^{het}$	4	249	4	7.8	0.5	6.8	1.0	5.8	2.1	1.0	2.0	1.0	1.2
$Trp53^{null}$; $N1\text{-}ICD^{het}$	9	116	8	65.4	25.8	55.0	21.9	38.9	15.6	16.1	8.6	10.4	6.1
$Trp53^{null}$	41	120	32	39.7	18.3	39.3	18.6	32.2	15.1	7.2	5.4	0.3	0.7
$Trp53^{het}$; $N1\text{-}ICD^{het}$	9	199	9	38.4	21.7	30.0	19.3	19.6	11.3	10.4	12.4	8.4	4.4

Supplementary Table 1. Descriptive statistics of the Tam→ENU cohort. Tumour counts are shown as the mean number of tumours per cohort (n = number of mice). Median survival (surv) is reported in days (n = number of mice). sd, standard deviation; proxSI, proximal small intestine; distSI, distal small intestine.

Cohort	n(Surv)	Surv	n(count)	Intestine	proxSI	distSI	Colon	sd Intestine	sd proxSI	sd distSI	sd Colon
Apc^{het}	2	356	5	10	NA	NA	NA	5.5	NA	NA	NA
Kras^{G12D}	7	361	7	3.6	3.3	0.3	0	3.4	3.1	0.5	0
Fbxw7^{null}	11	429	3	0.7	0	0.7	0	1.2	0	1.2	0
Pten^{null}	2	496	2	0	0	0	0	0	0	0	0
Trp53^{null}	5	614	5	2.4	2	0.2	0.2	1.5	1.2	0.4	0.4

Supplementary Table 2. Tamoxifen-only cohort descriptive statistics. Tumour counts are shown as the mean number of tumours per cohort (n = number of mice). Median survival (surv) is reported in days (n = number of mice). sd, standard deviation; proxSI, proximal small intestine; distSI, distal small intestine.

Abl1	Axin2	Cdx2	Ect2l	Flt4	Ifna5	Keap1	Mrps31	Pak3	Prg4	Slit2	Tlr4
Acan	Axl	Cep290	Eed	Fn1	Ifna6	Kel	Msh2	Palb2	Prkar1a	Smad2	Tmprss2
Acvr1	B2m	Cftr	Egfr	Foxa1	Ifnar1	Kit	Msh3	Pan3	Prkcd	Smad3	Tnfaip3
Acvr1b	Bap1	Chd3	Eif4a2	Foxa2	Ifnb1	Klf4	Msh6	Park2	Ptch1	**Smad4**	Tnfrsf14
Acvr2a	Bard1	Chd6	Eif4e	Foxl2	Ifne	Kmt2a	Msr1	Parp1	**Pten**	Smarca4	Top1
Adam29	Baz2b	Chd9	Eif4g1	Foxo1	Ifngr1	Kmt2c	Mst1r	Pax5	Ptgs1	Smarcb1	Tpte
Adamts18	Bcl11a	Chek2	Eif4g3	Foxp1	Ifngr2	Kmt2d	Mtor	Pbrm1	Ptpn11	Smarcd1	Traf6
Adar	Bcl2	Cic	Elf3	Fryl	Ifnlr1	Kmt2e	Mutyh	Pcbp1	Ptprb	Smo	Traf7
Afdn	Bcl2l1	Clcc1	Ep300	Fsip1	Ifrd1	Knstrn	Mx1	Pcdh18	Ptprd	Snx31	Traip
Aff4	Bcl6	Cltc	Epcam	Fubp1	Ifrd2	**Kras**	Myb	Pcdha2	Ptprk	Socs1	**Trp53**
Ahctf1	Bcl9l	Cnot1	Epha3	Fyn	Ift80	Larp4b	Myc	Pcdhb12	Ptprs	Sos1	Trp63
Ahnak	Bclaf1	Cntfr	Epha5	Gabra6	Igf1r	Lats1	Mycl	Pcdhga1	Ptprt	Sos2	Trpa1
Akap9	Bcor	Col11a1	Epha7	Gata1	Igf2	Lats2	Mycn	Pced1b	Rac1	Sox10	Trrap
Akt1	Birc3	Col1a1	Ephb1	Gata2	Ikbkb	Lepr	Myod1	Pdcd1	Rad21	Sox17	Tsc1
Akt2	Blm	Col2a1	Erbb2	Gata3	Ikbke	Leprot	Nbn	Pdgfra	Rad50	Sox2	Tsc2
Akt3	Bmpr1a	Col6a3	Erbb3	Gata6	Ikzf1	Lnpep	Ncoa3	Pdgfrb	Rad51c	Sox9	Tshr
Alk	Bmpr2	Col9a2	Erbb4	Gli1	Il10rb	Lrp6	Ncor1	Pgr	Rad52	Spen	Tshz3
Alox12b	Bptf	Cop1	Ercc2	Gna11	Il13ra1	Lrpprc	Ndufb9	Phox2b	Raf1	Spop	U2af1
Alpk1	**Braf**	Creb3	Ercc3	Gnaq	Il20ra	Lsamp	Negr1	Pias1	Ranbp17	Spred1	Ubr5
Amer1	Brca1	Crebbp	Ercc4	Gnas	Il21r	Lztr1	Nf1	Pik3c2b	Rapgef5	Spta1	Uggt2
Ank3	Brca2	Crkl	Ercc5	Gps2	Il31ra	Magi2	Nf2	Pik3c2g	Rara	Src	Usp9x
Ankrd11	Brd4	Crlf2	Erg	Grb2	Il3ra	Malt1	Nfatc4	Pik3c3	Rasa1	Stag2	Vhl
Ankrd17	Brd7	Crtc3	Errfi1	Grin2a	Il5ra	Map2k1	Nfe2l2	Pik3ca	Rasa2	Stat1	Vim
Ankrd35	Brip1	Csde1	Esr1	Gsk3b	Il7r	Map2k2	Nfib	Pik3cb	Rassf2	Stat2	Wasf3
Aox1	Brwd1	Csf1r	Etv1	H3f3a	Inha	Map2k4	Nicn1	Pik3cd	Rb1	Stat3	Wnk1
**Apc**	Bub1b	Csf2ra	Etv6	H3f3b	Inhba	Map3k1	Nipbl	Pik3cg	Rbm10	Stat4	Wt1
Aqr	Camta1	Csf3r	Ezh2	Hdac9	Inpp4a	Map3k13	Nkx2-1	Pik3r1	Rbm39	Stk11	Xiap
Ar	Card11	Csmd1	F5	Hdlbp	Inpp4b	Map3k14	Nkx3-1	Pik3r2	Recql4	Stk19	Xpo1
Araf	Casp8	Ctcf	Fam46c	Hgf	Insr	Map3k4	Notch1	Plcb1	Rel	Sufu	Xrcc1
Arfgef2	Cbfb	Ctla4	Fanca	Hist1h1c	Ireb2	Map3k5	Notch2	Plcg1	Reln	Suz12	Xrn1
Arhgap21	Ccdc28a	Ctnna1	Fancc	Hmcn1	Irf2	Map3k9	Notch3	Plcg2	Ret	Svep1	Yap1
Arhgap29	Ccnd1	**Ctnnb1**	Fancd2	Hnf1a	Irf4	Mapk1	Notch4	Plk2	Rgs3	Syk	Yes1
Arhgap35	Ccnd2	Cux1	Fancf	Hnf4a	Irf5	Max	Npas3	Plppr2	Rhoa	Syne1	Zbtb20
Arhgef2	Ccnd3	Cxcr4	Fancg	**Hras**	Irf6	Mbd1	**Nras**	Pmaip1	Rictor	Tacc1	Zfp292
Arid1a	Ccne1	Cyld	Fanci	Hrnr	Irf9	Mbd4	Nsd1	Pms1	Ripk1	Taok1	Zfx
Arid1b	Cd274	Daxx	Fas	Hydin	Irs1	Mcl1	Nthl1	Pms2	Rnf43	Tbl1xr1	
Arid2	Cd79a	Dcc	Fat1	Icosl	Irs2	Mdc1	Ntn4	Polb	Robo1	Tbx21	
Arid5b	Cdc27	Ddr2	Fat2	Idh1	Itga4	Mdm2	Ntrk1	Pold1	Robo2	Tbx3	
Aspm	Cdc73	Ddx3x	**Fbxw7**	Idh2	Itga9	Mdm4	Ntrk2	Pole	Ros1	Tcf12	
Asxl1	Cdh1	Ddx58	Fcrl1	Ifi35	Itsn1	Mecom	Ntrk3	Polg2	Rps6ka4	Tcf4	
Asxl2	Cdh2	Dicer1	Fgf3	Ifi44	Jak1	Med12	Nudt1	Polq	Rps6kb2	Tcf7l2	
Atf6b	Cdk12	Dis3	Fgf4	Ifih1	Jak2	Med23	Nuggc	Polr2a	Rptor	Tdrd9	
Atm	Cdk4	Dnah17	Fgfr1	Ifit1	Jak3	Mef2b	Nup210l	Polr2b	Runx1	Tert	
Atp1b4	Cdk6	Dnajc11	Fgfr2	Ifit2	Jun	Men1	Nup93	Pom121	Rybp	Tet1	
Atp2a2	Cdk8	Dnmt1	Fgfr3	Ifit3	Kat6a	Met	Nup98	Ppm1d	Sdha	Tet2	
Atp8b1	Cdkn1a	Dnmt3a	Fgfr4	Ifitm1	Kcnq3	Mga	Nutm1	Ppp2r1a	Sec24d	Tff3	
Atr	Cdkn1b	Dnmt3b	Fh1	Ifitm2	Kdm5a	Mgmt	Ogg1	Ppp6c	Setbp1	Tgfbr1	
Atrx	Cdkn2a	Dock2	Flcn	Ifna1	Kdm5c	Mlh1	Olfml2b	Prdm1	Setd2	Tgfbr2	
Aurka	Cdkn2b	Dot1l	Flt1	Ifna2	Kdm6a	Mpl	Oxa1l	Prdm2	Sf3b1	Tgif1	
Axin1	Cdkn2c	E2f3	Flt3	Ifna4	Kdr	Mre11a	Pak1	Prex2	Shq1	Tjp2	

Supplementary Table 3. Sequencing gene panel. List of genes included in exome sequencing experiment (n = 585). Genes included in multiplexed amplicon array (n = 10) are coloured (red = full exon coverage, green = partial coverage).

Gene	n_syn	n_mis	n_non	n_spl	wmis_cv	wnon_cv	wspl_cv	pmis_cv	ptrunc_cv	pallsubs_cv	qmis_cv	qtrunc_cv	qallsubs_cv
Ctnnb1	3	159	0	0	18.492229	0	0	0.000000	0.177202	0.000000	0.000000	0.716905	0.000000
Apc	12	36	157	17	1.156227	71.748681	71.748681	0.537952	0.000000	0.000000	0.972847	0.000000	0.000000
Ntrk3	2	22	2	3	2.446815	4.784903	4.784903	0.002651	0.007315	0.000847	0.415459	0.716905	0.165217
Prdm2	5	10	4	2	0.590802	5.267809	5.267809	0.135587	0.002084	0.001199	0.811451	0.304759	0.175370
Ros1	15	46	7	6	1.521579	3.230546	3.230546	0.053196	0.001023	0.002387	0.689284	0.246227	0.243254
Ahnak	25	35	4	0	0.527840	2.221641	2.221641	0.003551	0.168846	0.002530	0.415459	0.716905	0.243254
Hrnr	4	10	0	0	0.378243	0	0	0.004986	0.021199	0.002911	0.416653	0.716905	0.243254
Col11a1	13	26	3	9	1.129196	3.317495	3.317495	0.636082	0.001263	0.005326	0.972847	0.246227	0.389457
Tpte	1	19	2	0	2.742688	1.538097	1.538097	0.001463	0.581136	0.006259	0.415459	0.846950	0.406822
Prkn	0	0	0	0	0	0	0	0.003523	0.243577	0.008001	0.415459	0.716905	0.468038

Supplementary Table 4. dNdScv output table. Top ten genes ranked for *q* value (allsubs_cv). Table shows the number (*n*) of substitutions of each class (syn = synonymous, mis = missense, non = nonsense, spl = splice). Maximum likelihood estimates (MLEs) of the dN/dS ratio for each gene, for missense (wmis), nonsense (wnon), and essential splice sites (wspl). *q*-values are displayed for each class (qmis_cv = missense, qtrunc_cv = nonsense + splice) or integrating all classes (qallsubs_cv). Per-gene p-values come from likelihood ratio tests comparing observed vs. expected mutation counts. q-values are those p-values adjusted using Benjamini–Hochberg FDR correction.

name	mle	cilow	cihigh
wmis	1.011742	0.967	1.058555
wnon	1.415212	1.286735	1.556518
wspl	0.968402	0.855925	1.095659
wtru	1.222232	1.128892	1.32329
wall	1.032835	0.987858	1.07986

Supplementary Table 5. Global dN/dS estimates**.** Maximum likelihood estimates for the dN/dS ratios across all genes with confidence intervals (cilow, cihigh) were calculated separately for each substitution class and for all non-synonymous (wall). Theta = 25.1.

Cohort	n(Survival)	Survival	n(count SI)	SI	proxSI	distSI	sd SI	sd proxSI	sd distSI
Apc^{het} (Tam>ENU)	5.0	78.0	3.0	966.7	772.7	194.0	267.5	418.1	188.9
Apc^{het} (ENU>Tam_10d)	5.0	89.0	5.0	320.0	183.6	136.4	202.6	99.7	110.0
Apc^{het} (ENU>Tam_30d)	5.0	111.0	5.0	109.4	76.2	33.2	32.2	21.7	18.7
Fbxw7^{null} (Tam>ENU)	14.0	64.0	11.0	156.9	103.9	53.0	40.9	17.2	26.1
Fbxw7^{null} (ENU>Tam_10d)	5.0	67.0	4.0	134.8	73.0	61.8	33.0	22.5	16.1
Fbxw7^{null} (ENU>Tam_20d)	3.0	101.0	3.0	83.0	40.0	43.0	20.2	16.5	5.3
Fbxw7^{null} (ENU>Tam_30d)	5.0	217.0	4.0	18.5	6.8	11.8	7.3	3.8	4.8
Kras^{G12D} (Tam>ENU)	10.0	37.0	3.0	388.0	352.3	35.7	126.8	105.4	22.7
Kras^{G12D} (ENU>Tam_10d)	12.0	82.0	11.0	57.5	45.7	11.8	32.3	23.0	13.3
Kras^{G12D} (ENU>Tam_30d)	4.0	137.0	4.0	11.0	9.3	1.8	7.0	5.7	1.5
Trp53^{null} (Tam>ENU)	41.0	120.0	32.0	39.3	32.2	7.2	18.6	15.1	5.4
Trp53^{null} (ENU>Tam_10d)	9.0	157.0	9.0	19.1	16.6	2.6	9.4	7.5	2.9
Trp53^{null} (ENU>Tam_30d)	11.0	163.0	11.0	11.0	9.8	1.2	4.5	4.1	0.9

Supplementary Table 6. ENU→Tam cohort descriptive statistics. Small intestine counts only (proximal and distal). Tumour counts are shown as the mean number of tumours per cohort (n = number of mice). Median survival is reported in days (n = number of mice). sd, standard deviation; proxSI, proximal small intestine; distSI, distal small intestine.

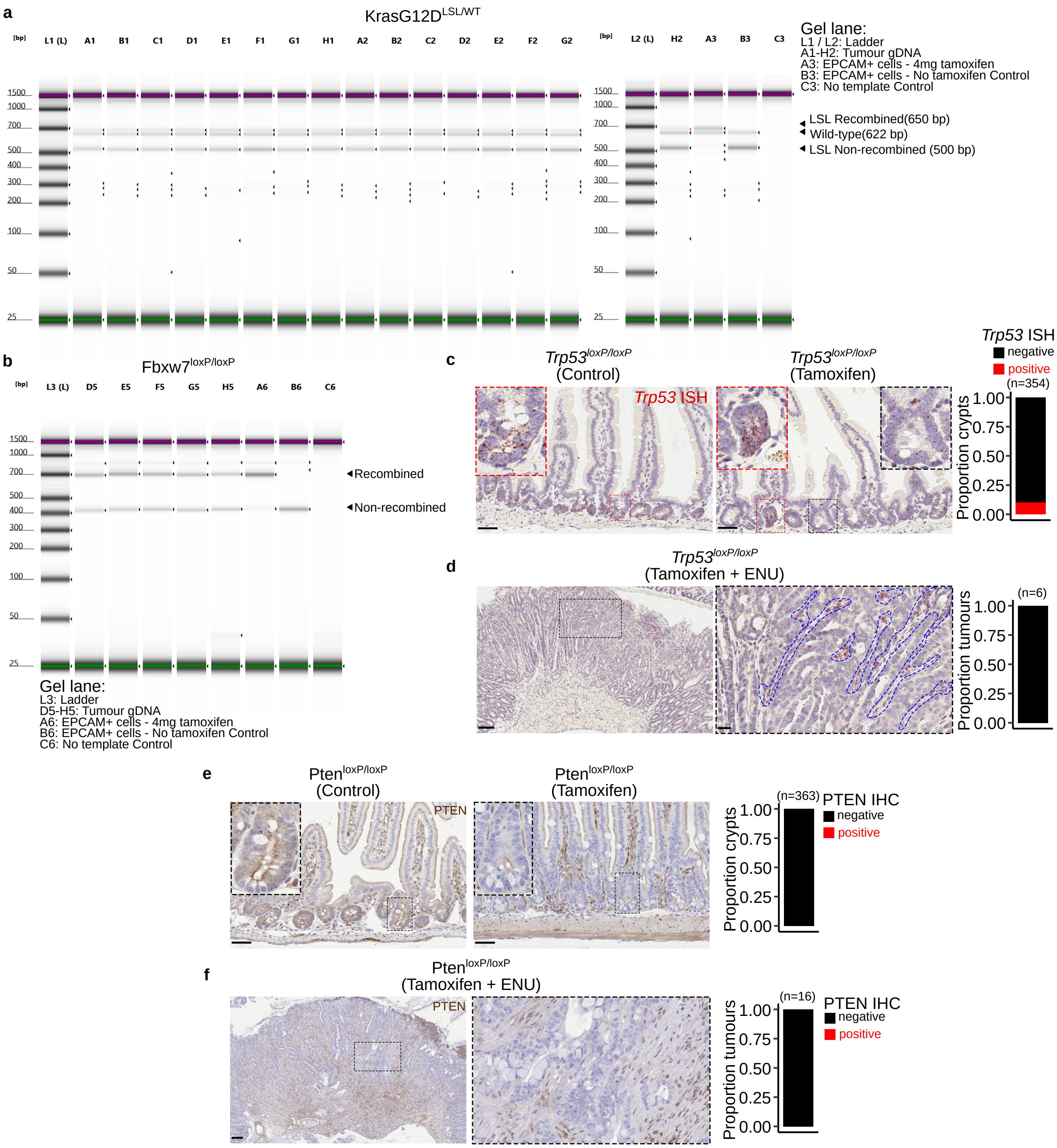

Supplementary Fig. 1. **Conditional Knock-out efficiency**. **a, b**, Genotyping PCR Tapestation™ gels for *Kras*^{G12D} (a) and *Fbxw7*^{null} (b) cohorts containing gDNA samples from a subset of sequenced tumours (*Kras*^{G12D} :16/211; *Fbxw7*^{null} :5/136), as well as EPCAM^{+} intestinal epithelial cells from control and tamoxifen-induced mice (n=1). **c**, Representative images of the small intestine, including higher magnification insets, from *Trp53*$^{loxP/loxP}$ mice showing *Trp53* RNAscope™ chromogenic (red) staining for control and tamoxifen-treated mice. The proportion of negative and positively stained crypts in tamoxifen-treated mice is presented as a column plot. Scale bar = 50 µm. **d**, Representative images of SI tumours from tamoxifen- and ENU-treated *Trp53*$^{loxP/loxP}$ mice. The inset shows a higher magnification view. The blue dashed line outlines *Trp53*$^{+}$ stroma. The proportion of negative and positively stained tumours is displayed as a column plot. Scale bars: 100 µm, 20 µm. **e**, Representative images of the SI, including higher magnification insets, of PTEN IHC staining from control and tamoxifen-induced *Pten*$^{loxP/loxP}$ mice. The proportion of negative and positively stained crypts in tamoxifen-treated mice is shown as a column plot. Scale bar = 50 µm. **f**, Representative image of SI tumours, with a higher magnification inset, from tamoxifen- and ENU-treated *Pten*$^{loxP/loxP}$ mice, showing negative PTEN staining in epithelial cells. The proportion of negative and positively stained tumours is presented as a column plot. Scale bar = 100 µm.

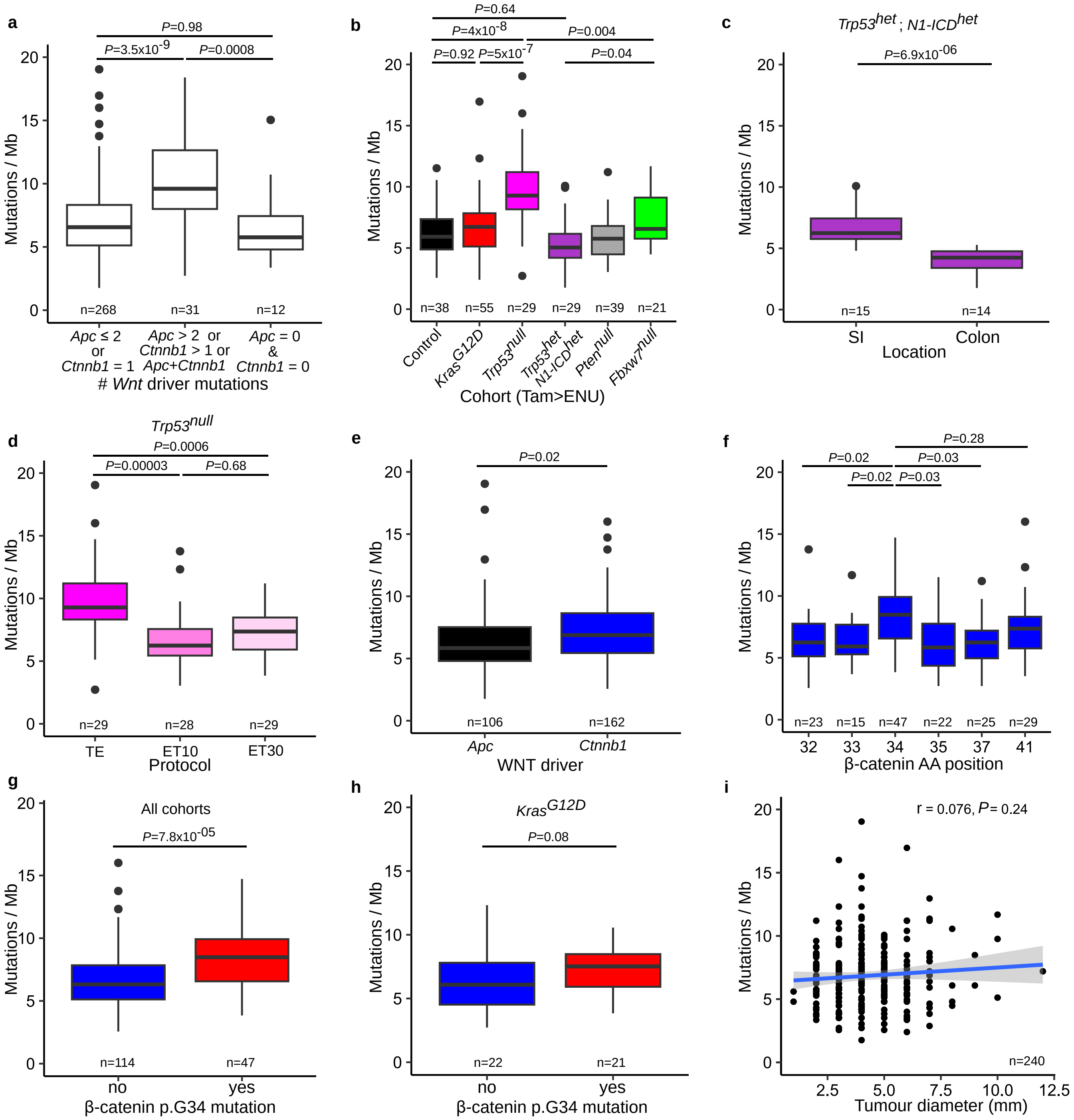

Supplementary Fig. 2. Mutation burden analysis. **a**, Mutational burden (mutations per Mb) in relation to number of driver mutations in *Apc* and *Ctnnb1* (Wnt drivers). Only tumours with *Apc* ≤ 2 or *Ctnnb1* = 1 were used for further analysis. **b,** Mutational burden in tumours from primed (TE) cohorts. **c**, Mutational burden relative to location in tumours from *Trp53*^{het}*;N1-ICD*^{het} cohort. **d**, Mutational burden relative to protocol (TE, ET10, ET30) in *Trp53*^{null} cohorts. **e**, Mutational burden relative to Wnt driver. **f**, Mutational burden of tumours with β-catenin driver mutations (positions 32, 33, 34, 35, 37, 41). **g**, **h**, Mutational burden relative to β-catenin p.G34 mutation status in all (g) or *Kras*^{G12D} (h) cohorts. **i**, Mutational burden relative to tumour diameter for informative samples (mm, nearest integer). Regression line with 0.95 CI. Statistical analysis was assessed using a Pearson correlation coefficient (two-sided). *P*-values in (a, b, d, f) were generated using ANOVA followed by a Tukey HSD multiple comparison of means test (two-sided) with results displayed in selected cohorts. *P*-values in (c, e, g, h) were generated using a two-sample *t*-test (two-sided). All boxplots display median and interquartile range (IQR; box bounds), with whiskers extending to most extreme points (≤1.5 × IQR) and all datapoints.

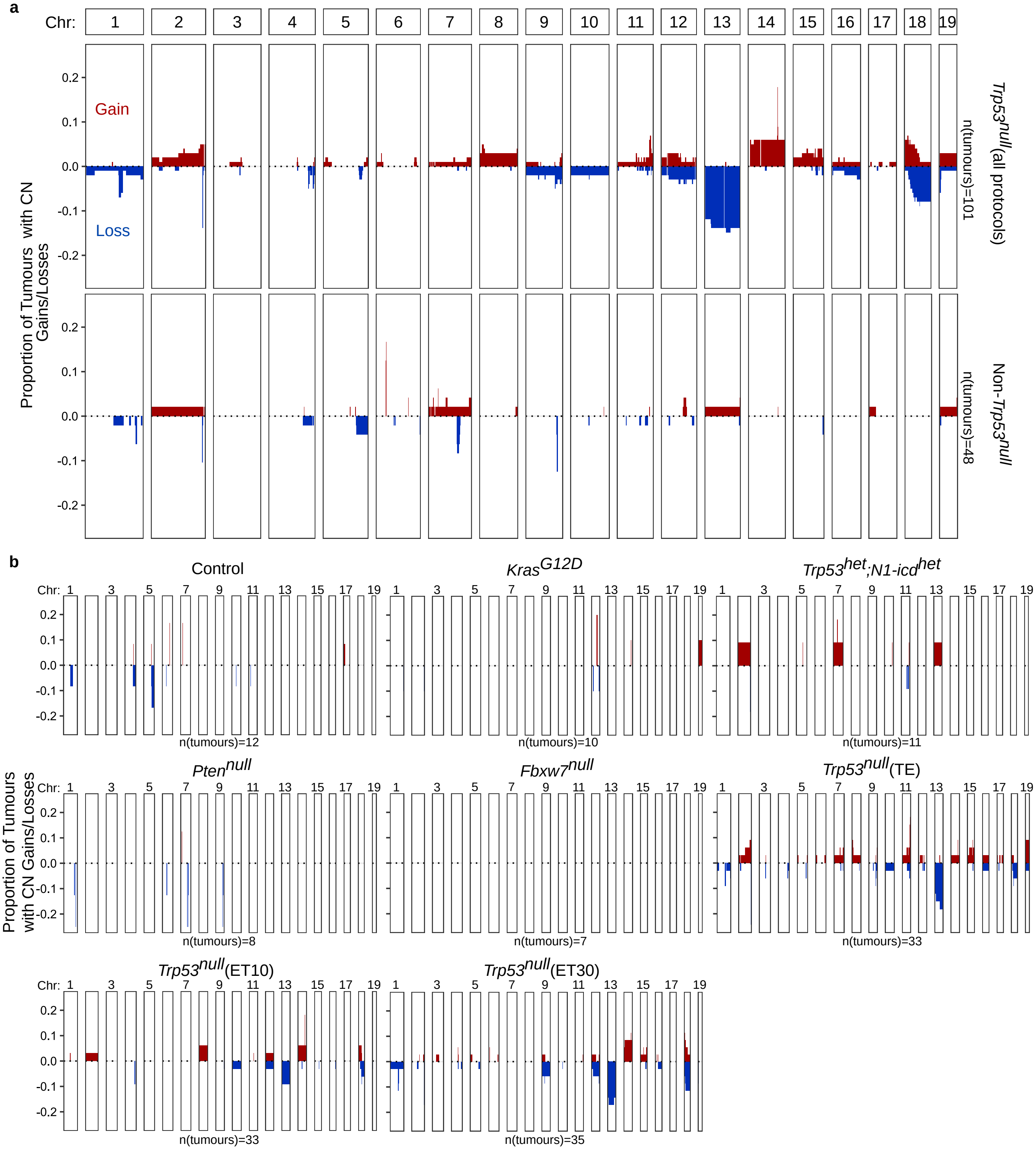

Supplementary Fig. 3. Copy number analysis in all cohorts. a, Frequency plot quantifying the proportion of tumours with copy number (CN) gains (red) and losses (blue), comparing high CN variation *Trp53*^{null} cohorts (all protocols: TE10, ET10, and ET30) with low copy number variation cohorts (control, *Kras*^{G12D}, *Fbxw7*^{null}, *Pten*^{null}, *Trp53*^{het};*N1-icd*^{het}). **b**, Frequency plot for each individual cohort from panel (a).

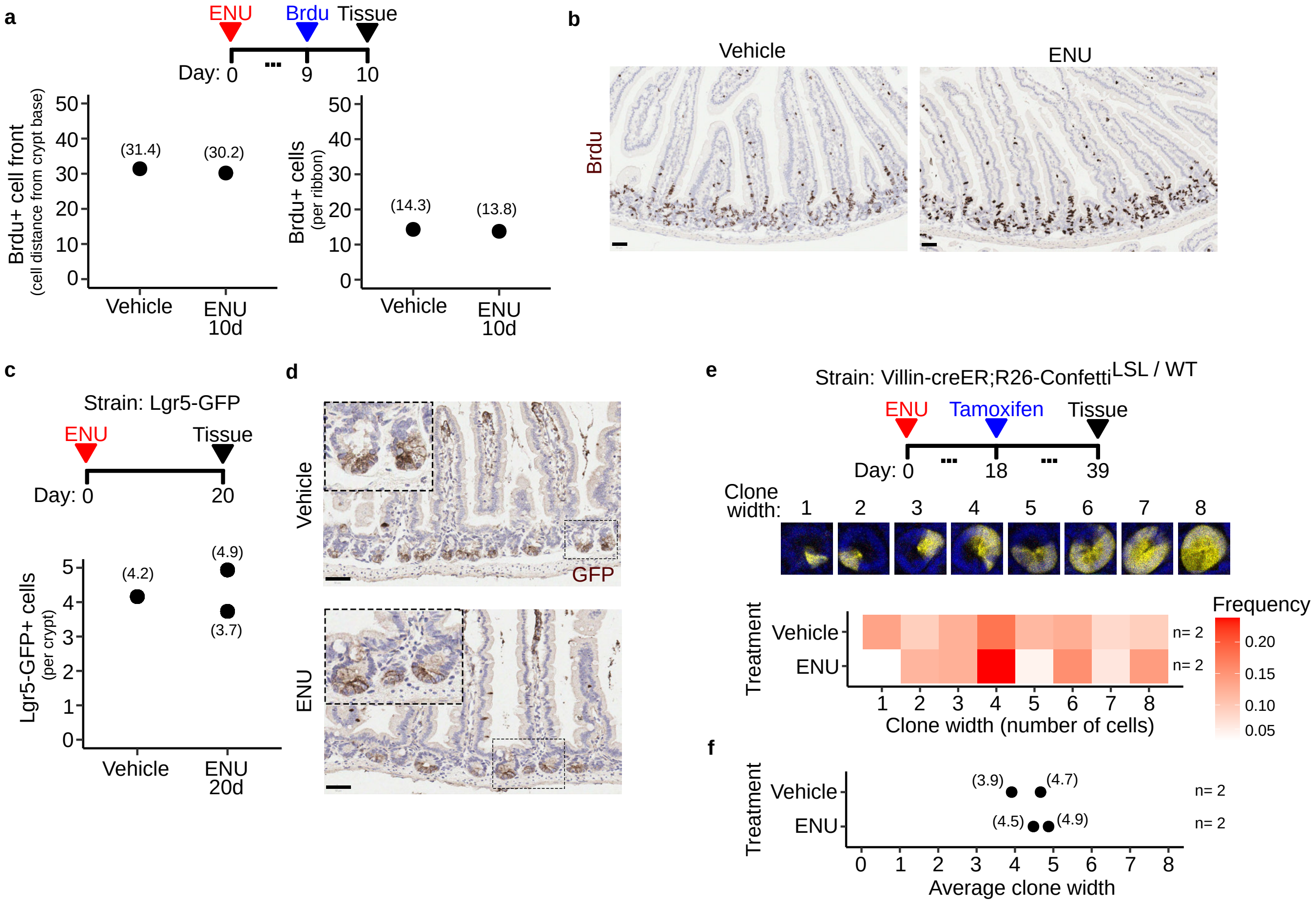

Supplementary Fig. 4. **Long term effects of ENU injection**. **a**, BrdU⁺ cell front and BrdU⁺ cells in relation to treatment (10 days post-ENU). Each data point represents BrdU⁺ measurements from one crypt-villus column. *n* = 1 mouse per cohort. **b**, Representative BrdU IHC images corresponding to (a) for the proximal small intestine. **c**, Lgr5-GFP⁺ cells per crypt in relation to treatment. *n* = 1 mouse (vehicle), *n* = 2 mice (ENU). **d**, Representative GFP IHC images corresponding to (c). **e**, Heatmap showing YFP⁺ clone width relative frequency in relation to treatment 21 days post-tamoxifen induction. **f**, Average clone width in relation to treatment. Brackets indicate mean values. Scale bar = 50 µm.

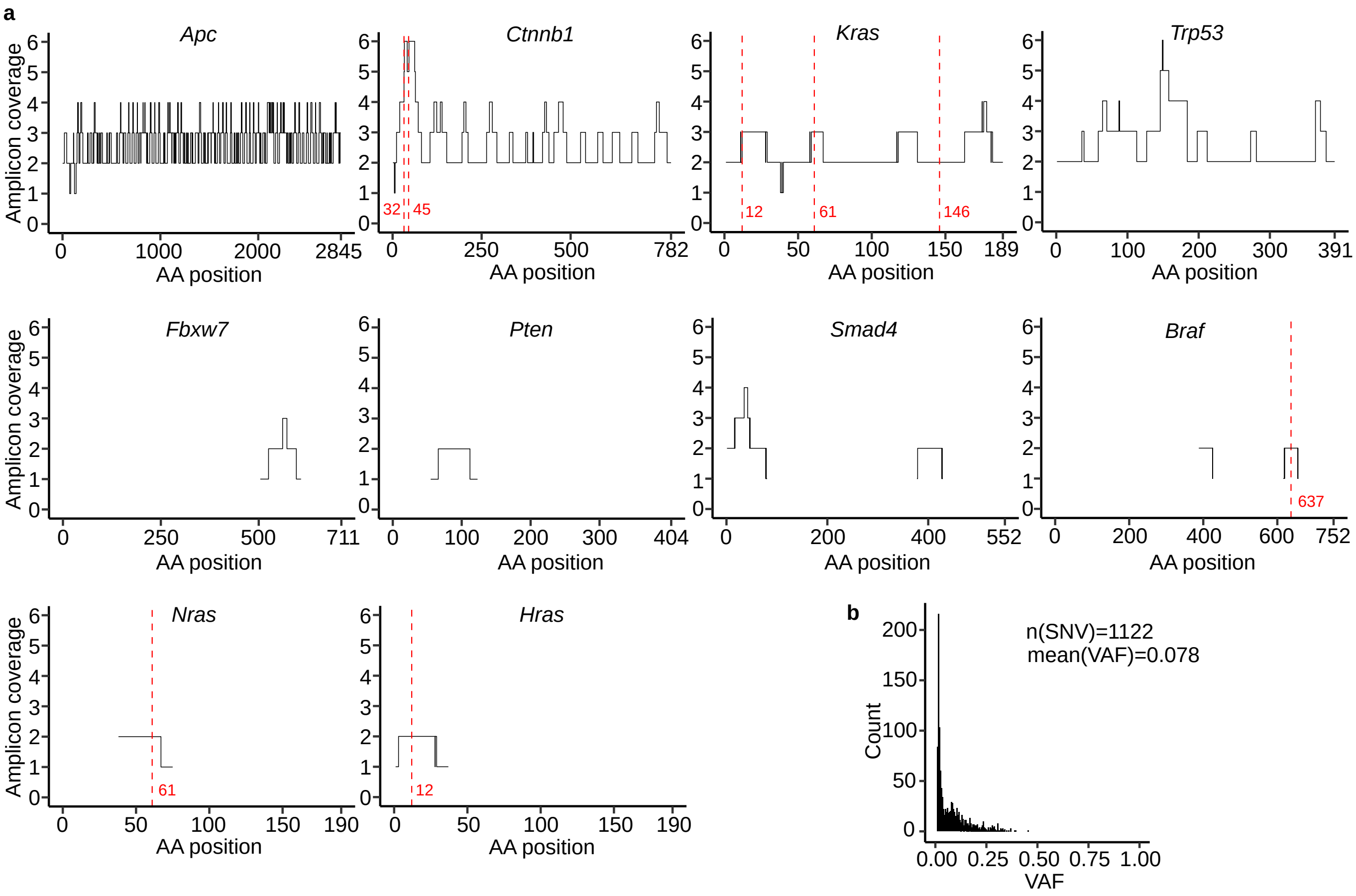

Supplementary Fig. 5. Amplicon-seq coverage and allele frequencies. a, Number of amplicons covering each gene's aa coding position in relation to its length. Dashed red lines mark mutational hot spot regions in *Ctnnb1*, *Kras*, *Braf*, *Nras*, and *Hras*. **b**, Variant allele frequency (VAF) histogram for each SNV called in tumour samples. *n*(SNV) and mean VAF displayed.

a

Control (DAPI+ ; Epcam-AF647-)

b

Experimental (DAPI+ ; Epcam-AF647+)

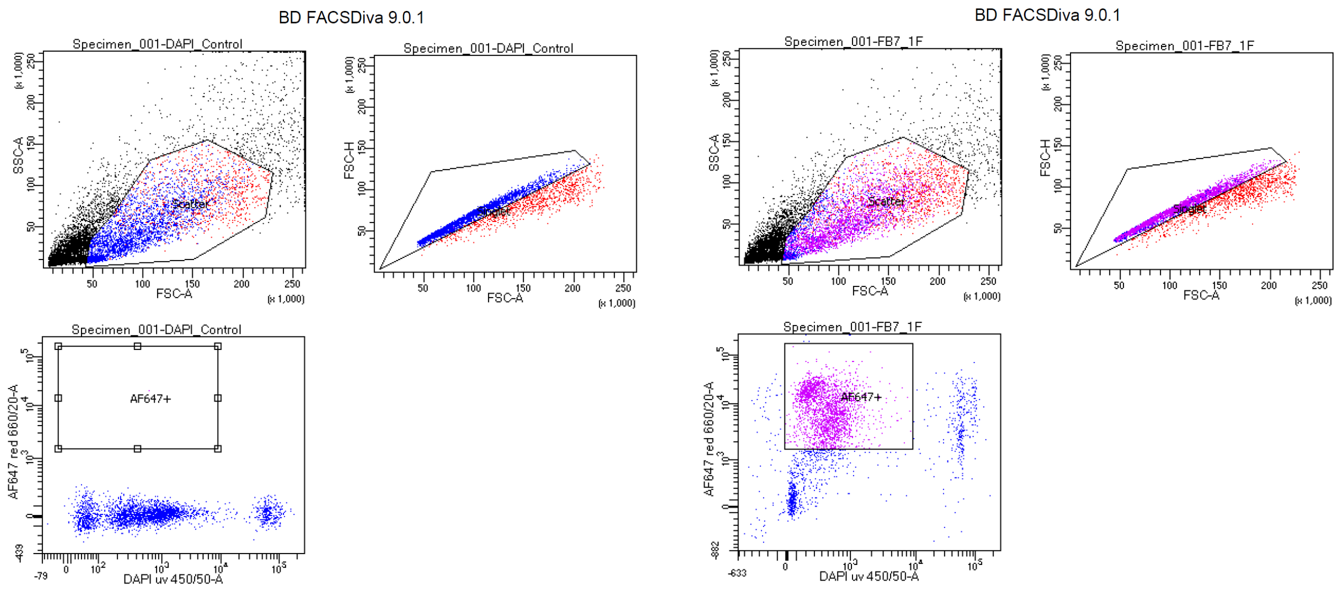

Tube: DAPI_Control

Population	#Events	%Parent	%Total
All Events	10,000	####	100.0
Scatter	3,968	39.7	39.7
Singlet	3,014	76.0	30.1
AF647+	1	0.0	0.0

Tube: FB7_1F

Population	#Events	%Parent	%Total
All Events	10,000	####	100.0
Scatter	4,538	45.4	45.4
Singlet	3,418	75.3	34.2
AF647+	2,312	67.6	23.1

Supplementary Fig. 6. FACS gating strategy. a, Gating strategy for control cells (DAPI+ and Epcam_AF647-). **b**, Gating strategy for experimental cells (DAPI+ and Epcam_AF647+).
