# Supplementary material for: Decay of driver mutations shapes the landscape of intestinal transformation
Source: Nature. 2025 Dec 3;649(8097):729–38. doi: 10.1038/s41586-025-09762-w (PMC12804087; doi:10.1038/s41586-025-09762-w)
Supplement: Supplementary file 2 — Reporting Summary [file 41586_2025_9762_MOESM2_ESM.pdf]

nature portfolio

Corresponding author(s): Douglas J Winton

Last updated by author(s): Oct 6, 2025

Reporting Summary

Nature Portfolio wishes to improve the reproducibility of the work that we publish. This form provides structure for consistency and transparency in reporting. For further information on Nature Portfolio policies, see our Editorial Policies and the Editorial Policy Checklist.

Statistics

For all statistical analyses, confirm that the following items are present in the figure legend, table legend, main text, or Methods section.

n/a	Confirmed	
☐	☒	The exact sample size (*n*) for each experimental group/condition, given as a discrete number and unit of measurement
☐	☒	A statement on whether measurements were taken from distinct samples or whether the same sample was measured repeatedly
☐	☒	The statistical test(s) used AND whether they are one- or two-sided<br>*Only common tests should be described solely by name; describe more complex techniques in the Methods section.*
☐	☒	A description of all covariates tested
☐	☒	A description of any assumptions or corrections, such as tests of normality and adjustment for multiple comparisons
☐	☒	A full description of the statistical parameters including central tendency (e.g. means) or other basic estimates (e.g. regression coefficient) AND variation (e.g. standard deviation) or associated estimates of uncertainty (e.g. confidence intervals)
☐	☒	For null hypothesis testing, the test statistic (e.g. *F*, *t*, *r*) with confidence intervals, effect sizes, degrees of freedom and *P* value noted<br>*Give P values as exact values whenever suitable.*
☒	☐	For Bayesian analysis, information on the choice of priors and Markov chain Monte Carlo settings
☒	☐	For hierarchical and complex designs, identification of the appropriate level for tests and full reporting of outcomes
☐	☒	Estimates of effect sizes (e.g. Cohen's *d*, Pearson's *r*), indicating how they were calculated

Our web collection on statistics for biologists contains articles on many of the points above.

Software and code

Policy information about availability of computer code

Data collection	Microscopy and image analysis/scoring:<br>Leica SP5 PCS microscope with LAS X software (v2.8.0, Leica)<br>Leica Aperio AT2 slide scanner<br>Qupath (0.5.1)<br>Fiji/Image J (version 1.53t)<br><br>FACS:<br>BD FACS Aria SORP (BD Biosciences)<br><br>Amplicon design:<br>Standard BioTools D3 Assay Design software<br><br>Hybridization capture probe bait design:<br>SureDesign (Agilent)<br><br>Sequencing:<br>Illumina HiSeq 4000<br>Illumina NovaSeq6000<br>Illumina NovaSeqX

Analysis scripts including Hybridisation capture sequencing variant calling, ENU mutation signature inference, and GENIE CRC cohort analysis are available in GitHub https://github.com/fcLourenco/ENU_mutagenesis_decay_driver_mutations.

Data analysis	Data and statistical analysis were performed in RStudio mostly with R version 4.3.2.<br>Data visualization was performed<br><br>DNA-seq variant calling:<br>biobambam2 (v2.0.79)<br>BWA-MEM (v0.7.17)<br>Pisces (v5.2.10.49)<br>Ensembl Variant Effect Predictor (Ensembl release 96)<br>Amplicon-seq pipeline (https://github.com/crukci-bioinformatics/ampliconseq)<br><br>Identification of driver genes:<br>dNdScv package (v0.1.0)<br><br>Copy Number analysis (sWGS):<br>BWA (v0.7.17)<br>QDNAseq (v1.30.0)<br>Rascal (v0.7.0)<br><br>INCISE cohort variant calling:<br>GIA HOLMES pipeline (V1.3.1)<br>Bcl2fastq conversion software (Illumina, V2.19.1.403, V2.20.0.422 on C++)<br>Burrows-Wheeler Alignment (V0.7.15)<br>deepSNV/Shearwater (V1.22/V1.1.0, V1.22.0.5/ V1.1.0)<br>Pindel (V0.2.5b8-ww1)<br>CAVA(V1.2.2.ww1, V1.2.2.ww5)<br>vcf2maf (V1.6.22)<br>Ensembl VEP (V112)<br>BRASS (V5.3.3-ww10)<br>geneCN (V2.1)

For manuscripts utilizing custom algorithms or software that are central to the research but not yet described in published literature, software must be made available to editors and reviewers. We strongly encourage code deposition in a community repository (e.g. GitHub). See the Nature Portfolio guidelines for submitting code & software for further information.

Data

Policy information about availability of data

All manuscripts must include a data availability statement. This statement should provide the following information, where applicable:

- Accession codes, unique identifiers, or web links for publicly available datasets
- A description of any restrictions on data availability
- For clinical datasets or third party data, please ensure that the statement adheres to our policy

DNA sequencing data from hybridisation capture sequencing is archived at the European Nucleotide Archive (ENA) under accession PRJEB26559. DNA sequencing data from amplicon sequencing and shallow whole genome sequencing (tumour copy number analysis) is archived in NCBI BioProject under accession PRJNA1327837. DNA sequencing data from INCISE polyp cohort is archived in EMBL-EBI ArrayExpress under accession E-MTAB-15619. The AACR GENIE colorectal cohort data was accessed through the registry's public data releases (https://https://genie.cbioportal.org/study/summary?id=genie_public; version 15). Source data is provided with this manuscript.

Research involving human participants, their data, or biological material

Policy information about studies with human participants or human data. See also policy information about sex, gender (identity/presentation), and sexual orientation and race, ethnicity and racism.

Reporting on sex and gender	Patients recorded sex was defined by the entry in their electronic health record and their Community Health Index (CHI) identifier. No sex or gender specific analysis was undertaken or reported in this manuscript.
Reporting on race, ethnicity, or other socially relevant groupings	No data on race, ethnicity or other socially relevant or vulnerable groups was collected or is reported in this manuscript.
Population characteristics	The patient cohort consists of 775 patients aged between 50 and 74 years. Patients who underwent polypectomy at bowel screening colonoscopy in NHS Greater Glasgow and Clyde between 2009 and 2016. Patients with colorectal cancer, past history of colorectal cancer, diagnosed polyposis or colorectal cancer predisposition syndrome and inflammatory bowel disease were not included.
Recruitment	Data including demography, clinical characteristics, endoscopy report results and histopathological data were collected

Recruitment	retrospectively. Data were collected from electronic health records (Clinical Portal, Orion Health, Boston USA and Trakcare, Intersystem, Boston USA) , electronic endoscopy reporting software (Unisoft GI Reporting Software, v2.5, Unisoft Medical Systems, UK) and Telepath Laboratory Information Management System electronic pathology database and linked using the Community Health Index (CHI) unique identifier.
Ethics oversight	Ethical approval was obtained for the retrospective use of data (GSH/20/CO/002) and analysis of surplus diagnostic tissue without individual informed consent (22/WS/0020) following applications to Glasgow SafeHaven, NHS Greater Glasgow and Clyde and the West of Scotland Research Ethics Committee.

Note that full information on the approval of the study protocol must also be provided in the manuscript.

Field-specific reporting

Please select the one below that is the best fit for your research. If you are not sure, read the appropriate sections before making your selection.

☒ Life sciences ☐ Behavioural & social sciences ☐ Ecological, evolutionary & environmental sciences

For a reference copy of the document with all sections, see nature.com/documents/nr-reporting-summary-flat.pdf

Life sciences study design

All studies must disclose on these points even when the disclosure is negative.

Sample size	Formal sample size calculation and power analysis were not performed because no prior data or reliable effect size estimates were available for these novel transgenic mouse models combined with ENU mutagenesis. Instead, sample sizes were informed by preliminary pilot experiments while adhering to the 3Rs (Replacement, Reduction, Refinement) to minimize unnecessary animal use.
Data exclusions	No data was excluded from analysis with the exception of a small number of sequenced tumour samples that were found a posteriori to be non-diseased.
Replication	Each tumour was treated as a biological replicate given its unique genomic composition resulting from random ENU mutagenesis. Tumour burdens were reproducible and consistent across experimental mouse batches. Tissue sections from n = 14–173 tumours (depending on cohort) were stained and scored for invasive features and β-catenin status. For knockout efficiency, n = 6–16 tumours (depending on genetic background) were analysed by PCR or assessed by Trp53 RNA staining or Pten IHC. O6-ethyl-2-deoxyguanosine IHC was performed on multiple normal tissue sections from one mouse per experimental condition, consistent with findings we previously reported (Sadien et al., Nature 2024). BrdU, Lgr5-GFP, and confetti lineage-tracing staining were conducted on multiple intestinal regions in n<3 mice, with replication constrained by animal availability.
Randomization	Formal randomization was not performed, however, groups were partially randomized by co-housing control and experimental animals irrespective of genotype and treating them simultaneously. Cohorts included a balanced representation of both sexes.
Blinding	For animal welfare reasons, researchers were not blinded to mouse genotype during study and data collection. Blinding was performed for IHC section staining count.

Reporting for specific materials, systems and methods

We require information from authors about some types of materials, experimental systems and methods used in many studies. Here, indicate whether each material, system or method listed is relevant to your study. If you are not sure if a list item applies to your research, read the appropriate section before selecting a response.

Materials & experimental systems

n/a	Involved in the study
☐	☒ Antibodies
☒	☐ Eukaryotic cell lines
☒	☐ Palaeontology and archaeology
☐	☒ Animals and other organisms
☒	☐ Clinical data
☒	☐ Dual use research of concern
☒	☐ Plants

Methods

n/a	Involved in the study
☒	☐ ChIP-seq
☐	☒ Flow cytometry
☒	☐ MRI-based neuroimaging

Antibodies

Antibodies used	1. CTNB1 antibody, mouse IgG1, clone 14/Beta-Catenin, cat#610154, BD Biosciences.<br>2. O6-ethyl-2’deoxyguanosine antibody, rat IgG2b, clone ER6, Cat# SQX-SQM001.1, Squarix Biotechnology

3. GFP antibody (10µg/ml, chicken, ab13970, Abcam)
4. PTEN antibody (1:300, rabbit, 9559, Cell Signalling)
5. BrdU antibody (2.6µg/ml, sheep, ab1893, Abcam)
6. CD326 (EpCAM) AlexaFluor 647 antibody (1:2,000, 118210, Biolegend)

Secondary antibodies:
7. rabbit anti-rat (1:250, A110-322A, Bethyl Laboratories)
8. rabbit anti-mouse (1:1500, ab125913, Abcam)
9. rabbit-anti-chicken (1:500, 303-005-003, Jackson Labs)
10. anti-sheep (1:500, 313-005-003, Jackson Labs).

Validation

Antibodies were validated by our histology core facility using negative controls. The staining patterns in our manuscript confirm previous publications:
1. Mientjes et al. Formation and persistence of O6-ethylguanine in genomic and transgene DNA in liver and brain of lacZ transgenic mice treated with N-ethyl-N-nitrosourea. Carcinogenesis (1996).
2. Tateishi K, Omata M, Tanaka K, Chiba T. The NEDD8 system is essential for cell cycle progression and morphogenetic pathway in mice. J Cell Biol. 2001; 155(4):571-579. (Clone-specific: Immunofluorescence, Immunohistochemistry)

Animals and other research organisms

Policy information about studies involving animals; ARRIVE guidelines recommended for reporting animal research, and Sex and Gender in Research

Laboratory animals

Mus musculus. The majority of mouse cohorts are near-inbred C57Bl/6J with outbred chromosomal regions corresponding to knocked-in genes. All experiments used male and female mice of at least 6 and 8 weeks of age, respectively.
The following strains were used:
VillinCreERT2
Kras(lsl)G12D/wt (KrasG12D)
Pik3ca(lsl)H1047R/wt (Pik3caH1047R)
ApcΔ14/wt (Apchet)
Trp53Δ2-10/Δ2-10 (Trp53null)
PtenΔ5/Δ5 (Ptennull)
Fbxw7Δ5/Δ5 (Fbxw7null)
R26-Notch1ic/wt (N1-ICDhet)
Arid1aΔ8/Δ8 (Arid1anull)
R26-CAG-Brainbow2.1/Confetti (R26(lsl)Confetti/wt)
Lgr5-DTR-EGFP

Wild animals

No wild animals were used.

Reporting on sex

Male and female mice were used in all cohorts with different proportions. Sex based analysis for tumour burden and sequencing were not included and can be provided at request.

Field-collected samples

No field collected samples.

Ethics oversight

All animal experiments were performed in accordance with the guidelines of the UK Home Office under the authority of a project licence (PD5F099BE) approved by the Animal Welfare and Ethical Review Body at the CRUK Cambridge Institute, University of Cambridge.

Note that full information on the approval of the study protocol must also be provided in the manuscript.

Plants

Seed stocks

Report on the source of all seed stocks or other plant material used. If applicable, state the seed stock centre and catalogue number. If plant specimens were collected from the field, describe the collection location, date and sampling procedures.

Novel plant genotypes

Describe the methods by which all novel plant genotypes were produced. This includes those generated by transgenic approaches, gene editing, chemical/radiation-based mutagenesis and hybridization. For transgenic lines, describe the transformation method, the number of independent lines analyzed and the generation upon which experiments were performed. For gene-edited lines, describe the editor used, the endogenous sequence targeted for editing, the targeting guide RNA sequence (if applicable) and how the editor was applied.

Authentication

Describe any authentication procedures for each seed stock used or novel genotype generated. Describe any experiments used to assess the effect of a mutation and, where applicable, how potential secondary effects (e.g. second site T-DNA insertions, mosiacism, off-target gene editing) were examined.

Flow Cytometry

Plots

Confirm that:

☒ The axis labels state the marker and fluorochrome used (e.g. CD4-FITC).

☒ The axis scales are clearly visible. Include numbers along axes only for bottom left plot of group (a 'group' is an analysis of identical markers).

☒ All plots are contour plots with outliers or pseudocolor plots.

☒ A numerical value for number of cells or percentage (with statistics) is provided.

Methodology

Sample preparation	The first 10 cm of proximal small intestine from tamoxifen-induced mice was flushed in cold PBS, longitudinally opened, and cut into 0.5 cm segments. The segments were repeatedly passed through a 10 mL pipette using cold PBS until the solution ran clear, incubated in cold 5 mM EDTA/PBS for 30 min, washed, and resuspended in cold PBS. Crypt-enriched fractions were released by 5 × 5 s manual shaking cycles then pelleted and dissociated in 20 mL trypsin (1 × 0.05% EDTA, 25300054, Thermo Fisher Scientific) at 37 °C for 7 min with vigorous shaking every minute. After multiple washes in PBS/2% FBS, dissociated cells were resuspended in PBS/2% FBS before incubation with anti-mouse CD326 (EpCAM) AlexaFluor 647 antibody and addition of DAPI (10 µg/mL) to distinguish between live and dead cells.
Instrument	BD FACS Aria SORP (BD Biosciences)
Software	BD FACS DIVA 9.0.1 (BD Biosciences)
Cell population abundance	Epcam-AF647+ cells represented between 18-31% of parental population.
Gating strategy	Gating strategy shown in Supplementary Fig.6.

☒ Tick this box to confirm that a figure exemplifying the gating strategy is provided in the Supplementary Information.
